# A Variational Approach to the Analysis of Dissipative Electromechanical Systems

**DOI:** 10.1371/journal.pone.0077190

**Published:** 2014-02-28

**Authors:** Andrew Allison, Charles E. M. Pearce, Derek Abbott

**Affiliations:** 1 School of Electrical and Electronic Engineering, University of Adelaide, Adelaide, Australia; 2 School of Mathematical Sciences, University of Adelaide, Adelaide, Australia; National Microelectronics Center, Spain

## Abstract

We develop a method for systematically constructing Lagrangian functions for dissipative mechanical, electrical, and electromechanical systems. We derive the equations of motion for some typical electromechanical systems using deterministic principles that are strictly variational. We do not use any *ad hoc* features that are added on after the analysis has been completed, such as the Rayleigh dissipation function. We generalise the concept of potential, and define generalised potentials for dissipative lumped system elements. Our innovation offers a unified approach to the analysis of electromechanical systems where there are energy and power terms in both the mechanical and electrical parts of the system. Using our novel technique, we can take advantage of the analytic approach from mechanics, and we can apply these powerful analytical methods to electrical and to electromechanical systems. We can analyse systems that include non-conservative forces. Our methodology is deterministic, and does does require any special intuition, and is thus suitable for automation via a computer-based algebra package.

## Introduction and Motivation

It is a widely believed that the Lagrangian approach to dynamical systems cannot be applied to dissipative systems that include non-conservative forces. For example, Feynman [1] writes that “*The principle of least action only works for conservative systems—where all the forces can be gotten from a potential function.*” Lanczos [2], writes “*Forces of a frictional nature, which have no work function, are outside the realm of variational principles, while the Newtonian scheme has no difficulty in including them. Such forces originate from inter-molecular phenomena, which are neglected in the macroscopic description of motion. If the macroscopic parameters of a mechanical system are completed by the addition of microscopic parameters, forces not derivable from a work function would in all probability not occur.”* Lanczos [2], and also writes “*Frictional forces (viscosity) which originate from a transfer of macroscopic into microscopic motions demand an increase in the number of degrees of freedom and the application of statistical principles. They are automatically beyond the macroscopic variational treatment.”* These eminent people were justified in their opinions. In 1931, Bauer[3] proved a corollary, which states that “*The equations of motion of a dissipative linear dynamical system with constant coefficients are not given by a variational principle.”* Since then, various mathematical scientists have been trying to find ways around this problem. It is clear that dissipative forces present a problem to traditional Lagrangian analysis, which means that the Newtonian approach has historically had an advantage, particularly where dissipative forces are significant.

There are a number of formalisms for applying a Newtonian (force-based) approach to mixed electromechanical systems. The bond-graph approach is based on the systematic use of effort and flow variables. The work of Karnopp et al. [4] is important in this regard. We will employ some aspects of Karnopp's work, including the homomorphic mappings of variables between different systems. There are clear analogies between mechanical and electrical oscillators, and we make use of these.

The Newtonian approach has been dominant in practical discipline areas, such as mechanical engineering. In contrast, the Lagrangian approach, which is very elegant, has tended to dominate advanced physics texts. For example, the Hamiltonian approach dominates the subject of quantum mechanics. Penrose [5], refers to this paradigm as the “*magical Lagrangian formalism.”* He goes on to write that “*The existence of such a mathematically elegant unifying picture appears to be telling us something deep about our physical universe.”*

There are a number of more prosaic factors in favour of the Lagrangian approach, which include:

- In the Lagrangian formulation, forces of constraint do no work, and need not be considered in the analysis. It is often not necessary to calculate internal stresses or forces of reaction.
- Post [6] points out that it is easy to state the underlying physical laws in arbitrary, curvilinear coordinates. It is possible to use generalised coordinates that directly reflect the nature of the physical system.
- Noether's theorem tells us that, if the Lagrangian function possesses a continuous smooth symmetry, then there will be a conservation law associated with that symmetry [5]. For conservative systems, this leads to the laws of conservation of momentum and conservation of energy. These conservation laws essentially give us one integration of the laws of motion for free. For example we can calculate the final momentum, and the final energy of a system without the need to explicitly integrate the laws of motion.
- Lagrangian modelling of machines, automatically takes care of energy transfer between different components of a whole system. This prevents incomplete models, which give rise to errors and paradoxes, such as the problem of the Penfield motor [7]. We believe that Lagrangian modelling is a natural choice, where energy is exchanged between different types of storage elements, in such systems as: a moving wire in a magnetic field, the D'Arsonval moving-coil meter, or for electromechanical systems more generally.
- In the Hamiltonian formulation, only first derivatives are required, not second derivatives.
- Many quantum systems, such as the hydrogen atom, only have a few degrees of freedom, and a complete description of all the microscopic parameters is possible. This means that frictional forces may not even need to be considered.

Perhaps the strongest theoretical motivation for the Lagrangian approach is that it explicitly represents the symmetries of the underlying physical laws. Melia [8] writes: “*As we shall see, the sole motivation for using action principles is to improve our understanding of the underlying physics, with a goal of extracting additional physical laws that might not otherwise be apparent.”*

Prior to the work of Riewe [9], [10], there was no satisfactory method for completely including non-conservative forces into a variational framework. Riewe writes that “*It is a strange paradox that the most advanced methods of classical mechanics deal only with conservative systems, while almost all classical processes observed in the physical world are non-conservative.”* We regard the approach used by Riewe as the most satisfactory method for including non-conservative forces into a variational framework. In this paper we apply his approach, for mechanical systems, to the new areas of electrical and electromechanical systems. This is still a topic of active research. The fractional calculus of variations has recently been presented comprehensively by Malinowska and Torres [11].

The work of Dreisigmeyer and Young is also significant. In 2003 they published a paper on nonconservative Lagrangian mechanics, which made use of fractional integration and differentiation [12]. In 2004, they extended the pessimistic corollary of Bauer [13], to show that is is not possible to derive a single retarded equation of motion using a variational principle. They then went on to suggest that a possible way around the dilemma would be to use convolution products in Lagrangian functions, citing the work of Tonti [14]. In 2004, Dreisigmeyer and Young[15] published another paper on nonconservative Lagrangian mechanics, in which they derived purely causal equations of motion. They made use of left fractional derivatives.

In this paper, we provide recipes for constructing Lagrangian functions, and show (by example) how these techniques can be employed more generally. We believe that the Lagrangian approach naturally models energy exchange within complex machinery, where energy can be stored and transferred between many different forms, including: energy of inertia, elastic energy, frictional loss, energy of the magnetic field, energy of the electric field, and resistive loss. Our approach can be used to confer the advantages of the variational method of analysis to a wide range of electromechanical systems, including systems that suffer from dissipative loss.

### A short summary of the variational approach

We can denote a Lagrangian function for a system as 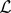, then we can specify the total *action* of the system as 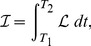
(1)where 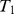 and 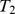 represent the boundaries of the closed time interval over which we wish to conduct our analysis. Equation 1 is referred to an *action integral.* It is a functional that maps functions, 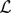, onto numbers, 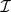. The Euler-Lagrange equation specifies a necessary condition for the first variation of the action integral to vanish, 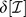  =  0. Suppose that the Lagrangian function includes references to a generalised coordinate, 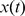, and to its first derivative 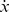 so 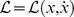, then the action is extremal when we choose 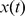 in such a way that the Euler-Lagrange equation is satisfied: 
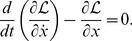
(2)


This is the same as saying that all first order variation of the action is zero, 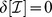. The Euler Lagrange equation is an ordinary differential equation that describes the dynamics of the system, in terms of the specified generalised coordinates, such as 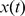.

For mechanical systems the Lagrangian is written in terms of energy functions, which are summed together with appropriate sign conventions. They typical symbols are kinetic energy of inertia, 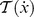, and potential (elastic or gravitational) energy, 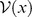. For these systems the Lagrangian function can be written as: 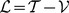. As we shall see, a classical example is a mass on a spring, where 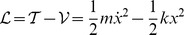.

We will use the notation of Gel'fand [16], who denotes a general 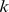
th order derivative as: 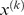. This is more versatile than the more traditional “dot” notation, of Newton. It is common for Lagrangian analysis to only consider integral derivatives, of low orders, of the generalised coordinates. For example, we might consider 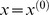, 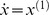 and possibly 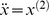. Gel'fand writes the generalised integer-order Euler-Lagrange equation a form that includes higher derivatives, and is equivalent to: 
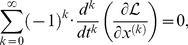
(3)where where 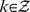, and where it is understood that 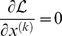, for values of 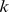 where the Lagrangian has no dependence on the 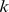
th derivative of the coordinate. The proof of Equation 3, can be obtained by repeatedly integrating by parts, and applying the du Bois-Reymond lemma. Proofs can be found in Gel'fand [16] and Smith [17].

Since the seminal work of Riewe [9], [10], a number of other authors have used his approach. These include Agrawal [18], Rabei [19], Frederico [20], Musielak [21], Elnabulsi [22], and Almeida [23]–[25]. Our main purpose here is to extend this work into the area of electrical circuits, and electromechanical systems.

#### Fractional Calculus

The indices of differentiation in The Euler Lagrange Equation 3 can be fractional, which leads to the formulation: 
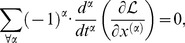
(4)where 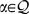, and where it is understood that 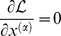, for values of 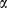 where the Lagrangian has no dependence on the 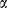
th derivative of the coordinate. The proof of this proposition depends on a fractional version of integration by parts, and is found in Riewe [9].

The theory of the fractional calculus has been well documented, and summaries can be found in Oldham et al. [26]. The topic of Fractional Calculus of Variations (FCV) has recently been presented, in an unified and complete way, by Malinowska and Torres [11]. We present a summary of basic results for convenience.

Fractional derivatives are not local unless 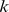 is an integer, which means that their value depends on a region around the point of evaluation. The choice of region is important. For engineering purposes, we only need to solve initial value problems, where time is between some initial time, such as 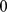, and a later time, 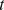. This is compatible with the left Riemann-Liouville fractional derivative, starting at zero: 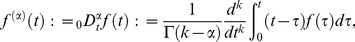
(5)where 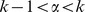. Of course in the case where 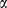 is an integer, and 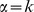, we have 
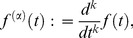
(6)which is the usual time-derivative. The definitions in Equations 5 and 6 are cited by Almeida [25], and we use them in this paper.

Fractional derivatives are not generally commutative, but in this paper we only need the semi-derivative, 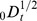, which is commutative 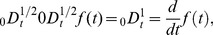
(7)together with the fact that fractional derivatives are the left-inverses of fractional integrals.

For engineering purposes, we often work with Laplace transforms. If we take the Laplace transform of Equation 5 then we obtain: 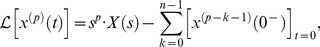
(8)where 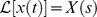. This equation can be used to define fractional derivatives for cases where the Laplace transform exists, although it may require initial values of *fractional* derivatives. There is a definition of fractional derivatives, due to Caputo, which only requires the initial values of derivatives with integral powers. This requires some degree of approximation. We do not explicitly use the Caputo definition in this paper.

We note that fractional derivatives can be complicated to work with, which can lead to human error. This is a limitation of the approach. We argue that the variational approach is worth the effort in cases where systems are compound, and exchange different types of energy between different parts of the system. In this case, the Lagrangian modelling is more likely to be complete, and not leave out essential terms. For engineering purposes, we are satisfied if our definitions give rise to correct ordinary differential equations of motion that are valid in a closed time-interval, 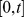.

## Discussion and Analysis

### A mechanical harmonic oscillator

We consider a common problem from classical mechanics, of a mass on a spring. This problem is widely used to define notation, and can be found in: Lamb [27], Goldstein [28], McCuskey [29], Resnick & Halliday [30], Whylie [31], Fowles [32], Feynman [1], Rabenstein [33] and Lomen [34], and many others.

**Figure 1:**
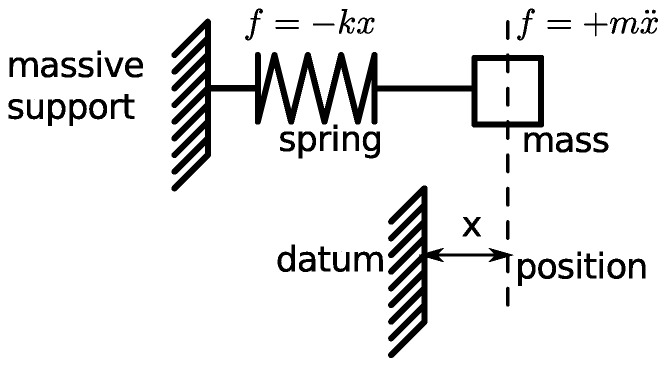
A mass on a spring. We consider the simple introductory problem of a mass, *m*, on a spring, with stiffness constant of *k*. The kinetic energy stored by the inertia of the mass is denoted by 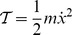. The elastic potential energy stored in the spring is denoted by 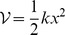. The independent coordinate is denoted by the position, *x*. The Lagrangian function is traditionally written as 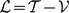, which can be written explicitly as 
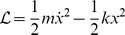
.

The mechanical harmonic oscillator consists of a mass, spring and massive support (or foundation). The complete system is shown in the schematic diagram in Figure 1. A mass, 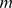, is attached to a spring, 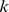, which is attached to a massive support. There is some difficulty with the schematic notation for the spring, 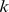, since the traditional schematic symbol for a spring resembles the traditional schematic symbol for a resistor. This creates problems if we need to represent both of these different objects in a single drawing. We have followed examples from Giesecke et al. [35]. In particular our symbol for the spring has a different aspect ratio to the symbol for the resistor, and the terminations at the ends are different. The position of the spring is measured relative to a datum position, which is in a fixed position relative to the massive support. Without any loss of generality we can choose the location of the no-load position of the mass, which gives a simple rule for the stored energy in the spring, 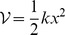.

The classical problem of a mechanical oscillator is shown in Figure 1. Together the mass and spring form a mechanical harmonic oscillator. Williams [36] specifies a Lagrangian for this physical system in terms of the single spatial coordinate, 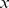, and writes: 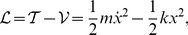
(9)where 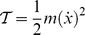, which is the kinetic energy in the inertia of the mass and 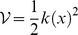 is the strain energy stored in the spring. The Lagrangian function, in Equation 9 is in a form where we can directly apply the Euler and Lagrange Equation to obtain: 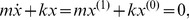
(10)which is the standard Ordinary Differential Equation (ODE) for this system. Equation 10 can be solved using a number of techniques, including the method of the Laplace transform, to obtain: 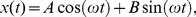
(11)where 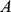 and 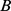 should be chosen in order to satisfy the initial conditions, and 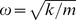 is the un-damped natural angular frequency of oscillation, in radians per second. In this completely un-damped case, 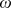 is also the resonant angular frequency.

### A homomorphic mapping

The example shown in Figure 1 is simple and well known, and lies completely within a mechanical problem domain. It is not immediately obvious how to extend this type of work to an electrical domain. We need a homomorphic mapping of variables that can relate different variables in different physical domains The mapping needs to relate the names of variables, as well as the set of permissible functions and operators that work on those variables. We use the mapping described in Karnopp et al. [4], which is summarised in Table 1.

**Table 1:** A homomorphic mapping due to Karnopp et al.

Concept	Mechanical	Electrical
Coordinate	displacement, *x*	charge, *q*
flow variable	velocity, 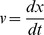	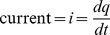
energy	energy 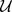	energy 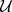
effort variable	force, 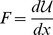	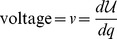
energy increment	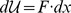	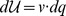
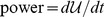	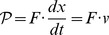	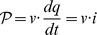
inertial element	mass, *m*	inductance, *L*
generalised momentum	momentum, 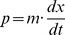	magnetic flux, 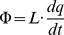
Newton's second law	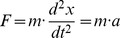	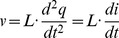
elastic element	stiffness constant, *k*	inverse capacitance, 1/*C*
Hooke's second law	*F* = −*k* · *x*	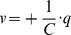
dissipative element	damping, *c*	resistance, *R*
frictional force	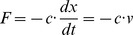	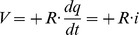
Joule's law	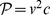	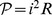
energy of inertia	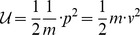	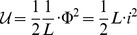
elastic energy	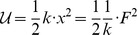	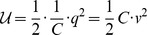

**A homomorphic mapping:** The names and purposes of the most important electromechanical dynamical concepts.

### An electrical harmonic system

**Figure 2:**
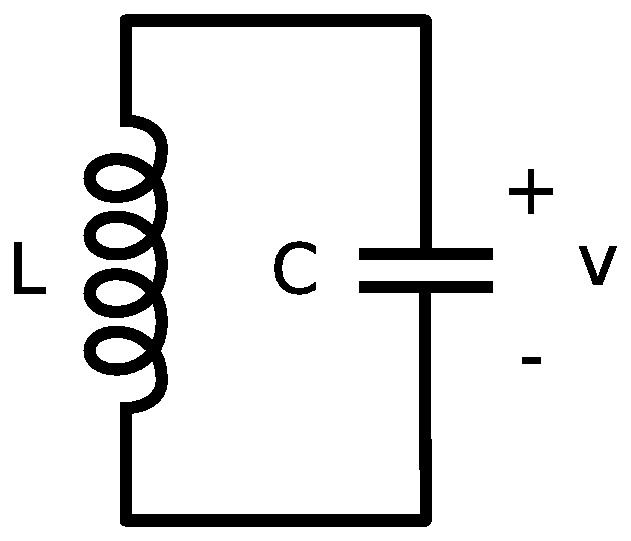
An LC electromagnetic harmonic circuit. We consider a capacitor, *C*, in parallel with an inductor, *L*. We consider the idealised case where there is no dissipative loss, or resistance *R*. The Lagrangian function can be written as 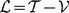, where the magnetic energy stored by the field of the inductor, denoted by 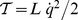, and the electrical potential energy stored in the capacitor is denoted by 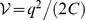, and *q*  =  *Cv* is the coordinate, which we interpret as the electrical charge that is transferred through the circuit.

If we place a capacitor, 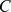 in parallel (and series) with an inductor, 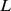, as shown in Figure 2, then the resulting system will form an electromagnetic harmonic oscillator.

If we temporarily ignore the presence of resistance, then we obtain the circuit in Figure 2 is the exact analogue of the mechanical system in Figure 1. In order to emphasise the homomorphic mapping between the mechanical and electrical domains, we map the Lagrangian function in Equation 9, using the homomorphic mapping in Table 1, to obtain
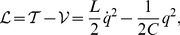
(12)where 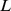 is the inductance, 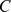 is the capacitance and 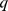 is the charge that is transferred through the circuit. We note that Equation 12 is a correct Lagrangian function for the circuit in Figure 2.

In Equation 12, we use the variable 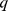 as a coordinate, in accordance with homomorphic mapping due to Karnopp. It is the more usual practice in electrical engineering to use the voltage across a capacitor, 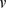, as though it were a generalised coordinate. Fortunately, it is possible to subject the Lagrangian function in Equation 12 to a Legendre transformation, of 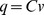, to obtain a new Lagrangian function that is consistent with the previous Lagrangian function (in terms of energy exchange), but uses the conventional coordinate of 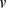 (rather than 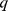). This new Lagrangian function is self-contained in the sense that the energy terms for inductor only include references to parameters that pertain to the inductor, and the energy terms for capacitor only include references to parameters that pertain to the capacitor. There are no cross-terms. If we impose this last condition then the Legendre transformation is unique and we obtain a new Lagrangian function: 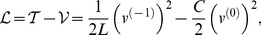
(13)where the independent generalised coordinate is now 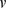. The function 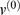 is the zeroth derivative of 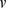, which is identical with 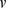. We can write 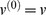. The function 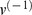 denotes the derivative of 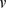 to the order of -1, which is equivalent to the integral of 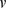. We can write 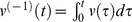.

#### Lagrangian terms for some common lumped electromechanical elements

We can see from the last example that electrical and mechanical systems can be mapped onto one another but some care has to be taken with regard to what we regard as a coordinate. The canonical choice for a massive particle is to regard the spatial position as the coordinate and to regard the generalised momentum as the other variable of interest. These choices are not arbitrary. The coordinate, 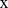, must be an exact differential, for example: 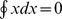 for all possible closed paths. In quantum mechanics, position, 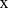, and momentum, 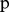, are conjugate variables. The relationship between 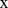 and 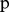 is a physical phenomenon, not just an arbitrary choice. Finally we know from classical mechanics that 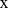 and 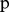 play a role in Liouville's theorem. See Reif [37] and Wannier [38], for example. Liouville's theorem would not apply in the same way if we were to describe particle motion in terms of force and velocity, rather than position and momentum. If we want to apply Liouville's theorem to a complicated electrical system with many degrees of freedom then we really need to use magnetic flux, 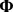, and electric flux, 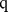, to describe each element of the system. This has been carried out in some specialist areas, such as Allison [39], but it is not common, and is not likely to become universal in the electrical engineering literature in the foreseeable future.

The conventional choice of electrical variables, voltage, 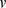, and current, 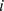 are of a fundamentally different type to the conventional choice of mechanical variables, 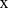 and 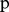. These incompatible conventions are not likely to change. The best solution seems to be that we should re-write the Lagrangian terms, using the conventional electrical variables, but to do so in such a way that the energy values are preserved, and all sign and phase relationships are consistent. These conversions are not physical laws. They have the same status as the conversion from degrees Fahrenheit to Kelvin, for example. The Lagrangian terms, for common electrical engineering lumped elements, are shown in Table 2 and Table 3. If we wish to include one of these devices in a system (and we wish to carry out Lagrangian analysis) then we only need to look up the relevant term in a table, and to include that term, in the Lagrangian function. No other special modelling or cognition is required. The process is direct enough to be able to be carried out by computer. In the interests of consistency with convention, we also include a table for the more familiar Lagrangian terms, in Table 4.

**Table 2:** Table of Lagrangian terms, in terms of current.

parameter	phasor	Lagrangian 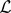	order, *k*	Euler-Lagrange
*inductance*, *L*	*I* = (−*j*/(*ωL*)) *V*	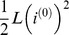	0	+ *Li* ^(0)^
*resistance*, *R*	*I* = (1/*R*) *V*	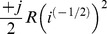	−1/2	+ *Ri* ^(−1)^
*capacitance*, *C*	*I* = (+*jωC*) *V*	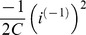	−1	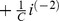

**Lagrangian terms, with current:** We list the common electrical lumped parameters, and compare the admittance with the corresponding term from the Lagrangian function. We also list the order of differentiation, *k*, and the corresponding term from the Euler-Lagrange equation. The phase direction of the Lagrangian term leads the phase direction of the admittance by 90°. This is equivalent to multiplying the Lagrangian term by +*j*. We can multiply the Lagrangian term by any constant that we like, as long as we do this consistently. If we were to remove the factor of +*j* then the Lagrangian terms and the admittances will have consistent phases, but all the Lagrangians will have new phases, and some of these will not be consistent with existing practice in mechanics. In this paper, we rigorously adopt the convention that is used in mechanics, which means that we do not use the sign convention that is common in electrical engineering.

**Table 3:** Table of Lagrangian terms, in terms of voltage, *v*.

parameter	Phasor	Lagrangian 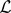	order, *k*	Euler-Lagrange
*inductance*, *L*	*V* = (*jωL*) *I*	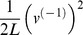	−1	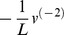
*resistance*, *R*	*V* = *RI*	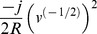	−1/2	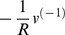
*capacitance*, *C*	*V* = −*jωCI*	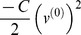	0	−*Cv* ^(0)^

**Lagrangian terms, with voltage:** We list common lumped electrical parameters, and compare the impedance with the corresponding term from the Lagrangian function. We also list the order of differentiation, *k*, and the corresponding term from the Euler-Lagrange equation. The phase direction of the Lagrangian term lags the phase direction of the impedance by 90°. This is equivalent to multiplying the Lagrangian term by −*j*. We can multiply the Lagrangian term by any constant that we like, as long as we do this consistently. If we were to remove the factor of −*j* then the Lagrangian terms and the admittances will have consistent phases, but all the Lagrangians will have new phases and some of these will not be consistent with existing practice in mechanics. In this paper, we rigorously adopt the convention that is used in mechanics, which means that we do not use the sign convention that is common in electrical engineering.

**Table 4:** Table of mechanical Lagrangian terms.

parameter	Lagrangian 3D	Lagrangian 1D	order, *k*	Euler-Lagrange
mass, *m*	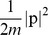	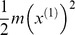	1	−*mx* ^(2)^
damping, *c*	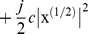	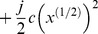	1/2	−*cx* ^(1)^
spring, *k*	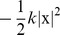	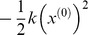	0	−*kx* ^(0)^

**Lagrangian terms for mechanical parameters:** We list the common lumped mechanical parameters. From left to right, we list the common symbol for the parameter, the Lagrangian for the 3D vector case (in terms of position or momentum), the Lagrangian for the 1D case (in terms of the position only), the order of differentiation employed, and the resulting them in the Euler-Lagrange Equation. We use the sign convention of Gel'fand and Fomin [16] for the terms of the Euler-Lagrange Equation. Some authors introduce an additional minus sign, in order to make all of these terms positive.

Finally, we note that some of the Lagrangian terms are imaginary, and that the resulting Lagrangian functions will, in general, be complex. We consider arithmetic operations to operate within the field of complex numbers, 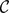. The traditional case, of real Lagrangian functions, is a special case of our more general formulation. Our formulation is consistent with the earlier work of Illert [40], who applied the concept of complex Lagrangian functions to the classical seashell problem.

### A damped mechanical harmonic system

**Figure 3:**
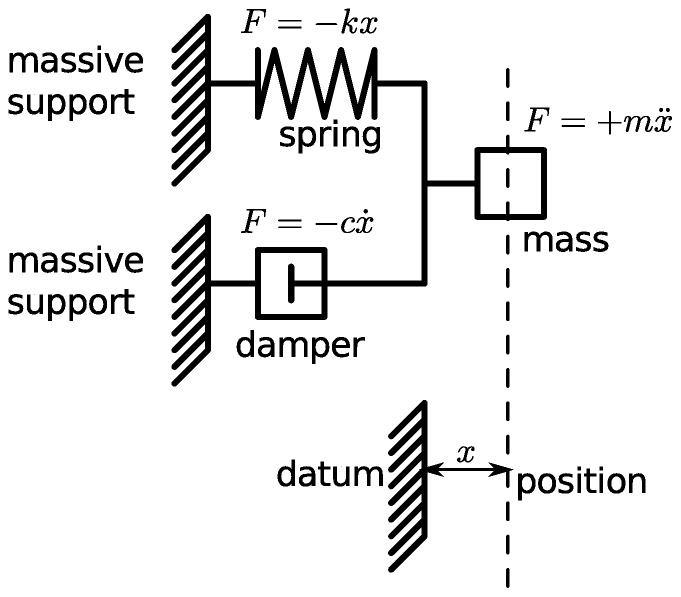
A damped mechanical harmonic oscillator. By introducing non-conservative, or dissipative, elements into a system we need to generalise our concept of potential. The Lagrangian for this system can be written as 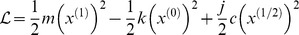. The additional term differs from the terms for the un-damped system in two key ways: the term is complex has an imaginary phase, of +*j*, and there is a fractional derivative of the coordinate, *x* ^(1/2)^.



We consider the damped mechanical oscillator, as shown in Figure 3, with mass, 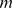, spring constant, 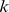, and coefficient of damping, 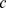. This problem is solved by Riewe [9]. In our case, we only need to take the Lagrangian terms from Table 4 and add them together to form the Lagrangian function for the system. This is shown as follows,

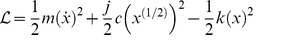


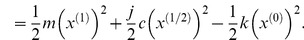
(14)


The resulting Euler-Lagrange equation can be assembled from the Euler-Lagrange terms in Table 4, or calculated directly, using Equation 4. The result is given by 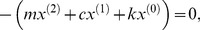
(15)which is the same result that we would obtain if we used a free body diagram and Newton's laws of motion.

### The use of constraints

The use of the calculus of variations to evaluate extremal functions, subject to constraints is described in a number of references, including Lanczos [2]. It is possible to regard perfect sources, of voltage or charge (or force or velocity), as constraints. This can simplify the working of some problems. We include an example here.

We consider the case of a purely resistive system. Jaynes [41] traces this problem back as far as Kirchhoff [42], and points out that the condition that no electric charge should accumulate at any point in a resistive material requires that 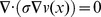, which is just the Euler-Lagrange equation stating that the production of Joule heat in a domain 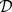, 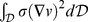 is stationary with respect to variations 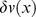 that vanish at the boundary of 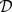. It should be noted that this variational principle applies only to strictly resistive circuits. It needs to be embedded into another theory, or extended if we have to model combined systems, which include stored energy.

Kirchhoff's voltage law is partly a matter of definition, but it is not arbitrary. It relates to thermodynamics in subtle ways. In a quasi-static situation where radiation is not significant voltage is just energy per unit charge, 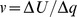. It is tempting to regard Kirchhoff's current law as a statement of the conservation of charge, but this is misleading. Even if we grant the continuity of charge, 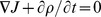 then it would still be possible to have accumulation of charge. The equivalent principles of “no accumulation of charge” and “minimum production of Joule heat in a domain” are ultimately statistical in nature, and are related to the second law of thermodynamics. This is discussed in Allison [39].

**Figure 4:**
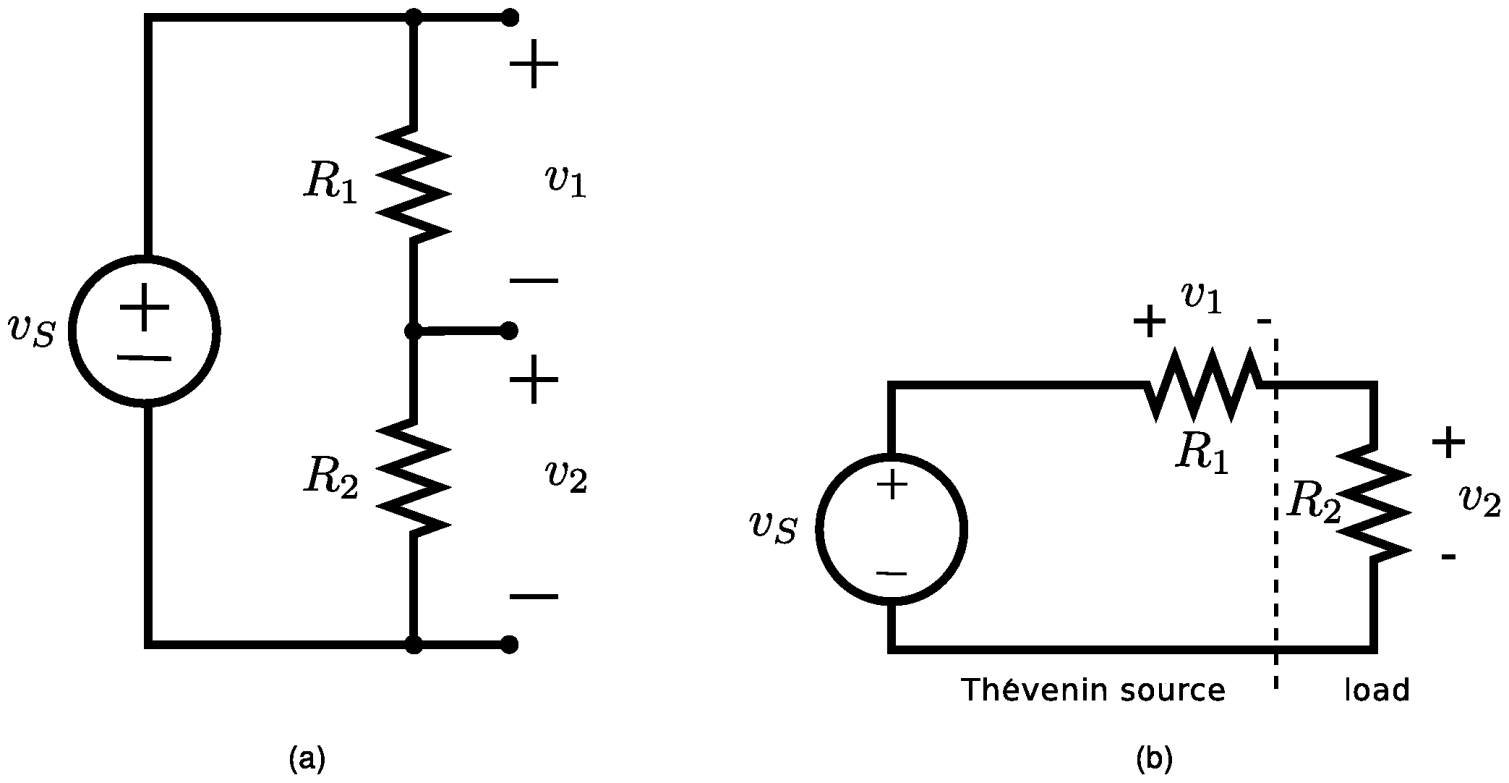
Two resistors in series. The voltage source, *v_S_* places a constraint on the voltages across the two resistors, *v*_1_ and *v*_2_. Kirchhoff's voltage law implies that Ψ  =  *v*_1_ + *v*_2_ − *v_S_*  =  0. We can regard the function Ψ as a function of constraint. Kirchhoff used a function that is equivalent to the dissipated power as a Lagrangian function. In modern notation we can write 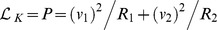. In order to ensure compatibility with other existing Lagrangian functions, we need to apply a transformation to obtain a new Lagrangian function of 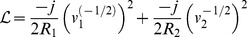. This Lagrangian function has been multiplied by a scalar of −*j*/2 and the order of differentiation has been reduced from *v* ^(0)^ to *v* ^(−1/2)^, which is equivalent to a half-integration of the Lagrangian function, or a full integration of the resulting Euler Lagrange equation.



In this paper, we consider the special case where the parameters are lumped into two resistors in series, as shown in Figure 4. To simplify the notation, we define a gradient operator, 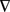, over variations with respect to the variational operators, 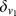 and 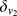, rather than partial derivatives, 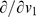 and 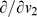. We can define

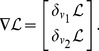
(16)This allows us to use the notation of the Lagrange multiplier to write down a necessary condition for a constrained optimum. If the independent variables, 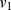 and 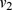 are constrained by a function of constraint 
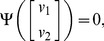
(17)then we can only obtain constrained stationary values of 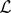 when 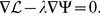
(18)


We apply this principle to the problem in the next section.

### A problem with two resistors

In Figure 4(a), the voltage source, 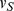 places a constraint on the voltages across the two resistors, 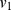 and 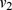. We can apply Kirchhoff's voltage law to the single mesh in this circuit to obtain 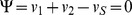, where 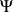 is a function of constraint. Gradients of this constraint function are needed in order to determine the constrained stationary functions of the system. Kirchhoff noted that the voltages in a resistive circuit, such as 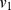 and 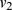, would arrange themselves in such a way as to minimise the dissipated heat energy, given by Joule's law. Jaynes points out that this is equivalent to defining a *“*Kirchhoff” Lagrangian function. For us, this takes the form 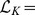 power 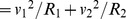. In order to ensure compatibility with other existing Lagrangian functions, we apply a Legendre transformation to obtain a new Lagrangian function 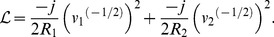
(19)


The resulting Lagrangian function, in Equation 19, can also be assembled from the Euler-Lagrange terms in Table 3. It is consistent. In Figure 4(b) we indicate that this situation is very common since it occurs whenever a linear source is connected to a resistive load.

We can use the principle of the Lagrange multiplier to obtain equations for the stationary functions, subject to constraints. We begin by calculating the individual variations: 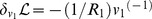, and 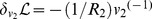, which leads to the following form for the gradient, 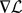, as 
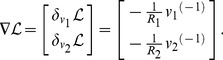
(20)


The function of constraint is 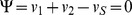, and we obtain the gradient of this as 
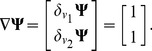
(21)


We can apply the principle of the Lagrange-multiplier to obtain 
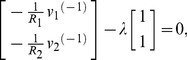
(22)for some constant complex number, 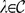. If we differentiate once, with respect to time, and multiply by 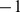, we obtain: 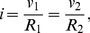
(23)where 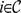 is a complex number. Since 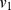, 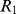, 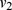 and 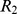 are all real we an infer that 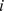 is real. If we consider Ohm's law then 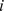 is a common current that is shared by both resistors. Equation 23 can also be obtained by using Kirchhoff's current law and by applying Ohm's law twice. Our main aims in presenting this last example are:

- to illustrate the use of constraints, with possible time and rate dependence,
- to demonstrate the utility of our extended Lagrange-multiplier notation,
- to provide a historical reference to the important work of Kirchhoff and Jaynes, and
- to resolve an apparent contradiction between the Lagrangian analysis of purely reactive systems which only have energy storage, and the Lagrangian analysis of purely resistive systems, which only have power dissipation. The reactive systems have Lagrangian terms that only depend on energy terms. The resistive systems have Lagrangian terms that only depend on power terms. Superficially, this appears to be a contradiction.

In order to examine the proper relationship between “energy” Lagrangian terms and “power” Lagrangian terms further, we next consider a mixed example, where the system contains both resistive and reactive elements, tied together with a constraint.

### A damped electrical harmonic system

**Figure 5:**
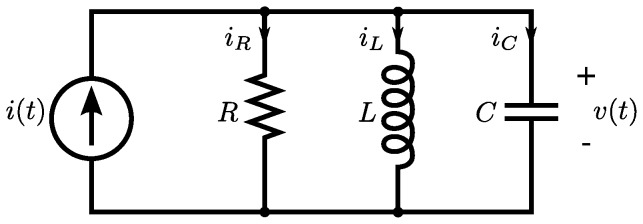
A parallel RLC circuit, with source. We can use the established rules to write the Lagrangian function for the circuit as: 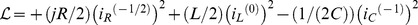. We also use Kirchhoff's current law to impose a constraint function of **Ψ**  =  *i_R_* + *i_L_* + *i_C_* − *i_S_*  =  0, and we use the principle of the Lagrange multiplier to obtain an ordinary differential equation to describe the dynamical behaviour of *v*(*t*).



We can use the terms in Table 2 to write the Lagrangian function for the circuit in Figure 5 as:

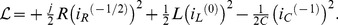
(24)We can use Kirchhoff's current law to impose a constraint function of 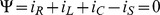. We can then use the techniques from the last section to obtain the solution to the problem of the dynamics of this circuit. We then find a functions, 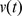, that give stationary values for the of the action, 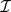, subject to the constraint, 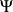. The principle of the Lagrange multiplier allows us to replace the optimising principle with a new constraint, 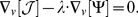
(25)


We can combine this new constraint with the original constraint, 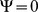, and use algebraic techniques to obtain an ordinary differential equation for the dynamics of the circuit.

We can use the Euler-Lagrange terms in Table 2 to write: 
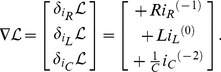
(26)


The function of constraint is derived from Kirchhoff's current law and can be written as 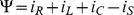, and we obtain the gradient of this as 
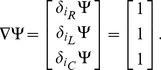
(27)


We can apply the principle of the Lagrange-multiplier to obtain 
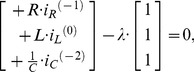
(28)


for some constant complex number, 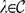, which leads to the result 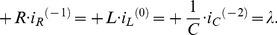
(29)


If we apply the constitutive laws for the three devices, and Kirchhoff's voltage law, three times, then we realise that we can interpret 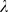 as the common voltage across all three components, 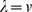. Of course, we could have obtained this result using more conventional circuit theory but the point here is that we have arrived at differential equations for the system in Figure 5 using purely variational techniques, and we have been able to model a complete electrical system that includes a dissipative element, 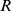.

### A ladder filter

**Figure 6:**
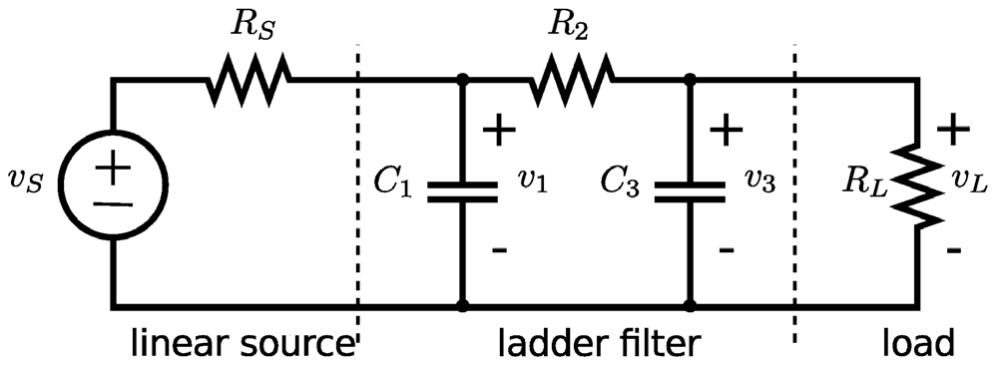
An electrical ladder-filter circuit. In this circuit, we make a careful choice of generalised coordinates, which allows us to avoid the explicit use of functions of constraint. We can use the established rules to write down the Lagrangian function for the circuit as shown in Equation 30. We apply the rules for the Euler-Lagrange equation to this Lagrangian function to obtain a pair of ordinary differential equations that describe the dynamics of this circuit.

The use of constraints can be a powerful technique, but it does add some extra complication to the analysis. It is often possible to make a careful choice of generalised coordinates, and avoid the need for constraints. We demonstrate this concept by analysing the ladder circuit in Figure 6.

If circuits have obvious regularity or symmetry, like the ladder circuit, then it is often possible to choose the coordinates in such a way that constraints are automatically obeyed. For the circuit in Figure 6, we can choose the state-variables as 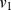 and 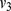. The conventional expression for the stored energy in the system can be written entirely in terms of these state variables as 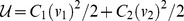. It is also possible to express all the voltages across the resistors in terms of 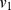, 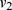 and 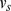, using Kirchhoff's voltage law. Kirchhoff's Voltage Law (KVL) does impose constraints on the system. We implicitly use KVL, and apply constraints, in the definition of the Lagrangian function, 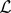. This means that we do not need to explicitly use a Lagrange multiplier technique. Our decision to use the state-variables as the independent coordinates of the system means that the Lagrangian function in Equation 30 takes a simple form and can be written down almost as quickly as the circuit can be drawn. Our choice also ensures that the final Euler-Lagrange equations are closely related to the state variable model, which could be obtained by using conventional circuit analysis. It is also clear, from the symmetry of ladder circuits, that we could extend this Lagrangian technique to ladders of arbitrary length and composition, as long as they were composed of components from Table 3.

The Lagrangian function can be written directly as: 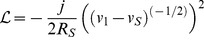


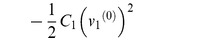


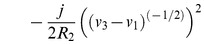


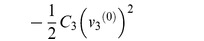


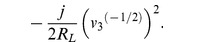
(30)


This Lagrangian function is completely composed of terms that can be found in Table 3. We can use the established rules to calculate the variations of the Lagrangian: 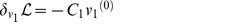


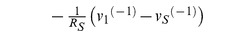


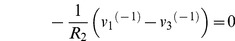
(31)and 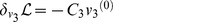


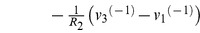


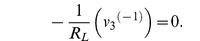
(32)


We can differentiate these variations, once with respect to time and rearrange the equations into the usual form of a state-variable model of the form 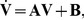
(33)where the time-rate of change is 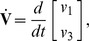
and the transition-matrix is 
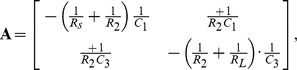
and the state-vector is 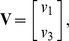
and the source-vector is 
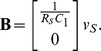



There is great advantage in noting the symmetries of any circuit that is being analysed, and choosing the generalised coordinates in a consistent manner. For example, we can exploit the symmetry of ladder circuits to extend our Lagrangian technique to ladder circuits of arbitrary length.

### A electromechanical problem, the D'Arsonval galvanometer

One of the great advantages of the Lagrangian approach is that it can be easily used to model devices that transduce energy between different forms. For example, an electric motor (or generator) transduces energy between electrical energy (in electric and magnetic forms) to, and from, mechanical energy (in kinetic and elastic forms).

**Figure 7:**
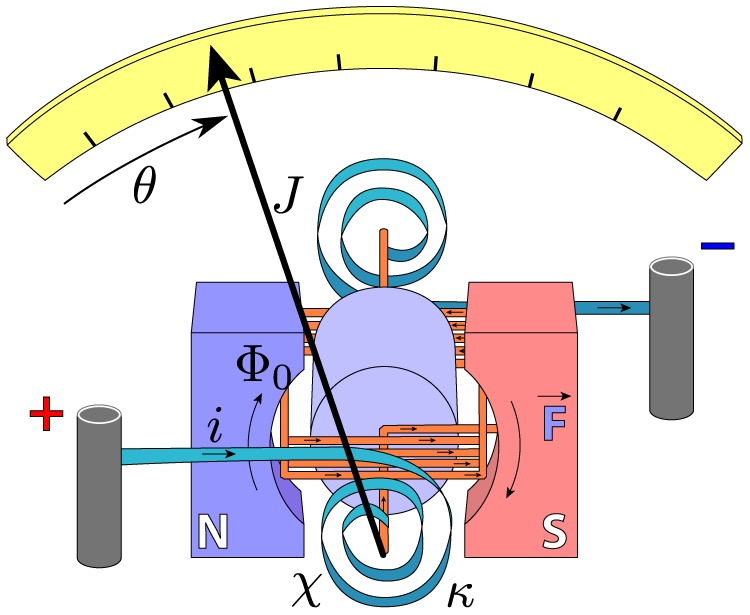
Physical layout of the D'Arsonval galvanometer. We model the essential features of the D'Arsonval meter as: the rotational moment of inertial of the coil *J*, the torsional spring constant, *κ*, the torsional damping constant, *χ* and the maximum magnetic-flux linked by the coil, **Φ**_0_. We use a linear model for the stored energy in the coil, 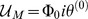. The Lagrangian function can be written in terms of these fundamental parameters. (Adapted from the Wikimedia commons.)



The simplest form of electric motor is a piece of wire, moving in a magnetic field. A short element of wire, 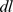, moving in a magnetic field will experience Lorenz force of 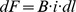, provided that the wire and the fields are orthogonal. The modern form of the moving-coil current meter is the result of a long line of development, which includes contributions from many people, including Oersted, Schweigger, Kelvin, D'Arsonval, Weston, and Ayrton. A typical physical meter is shown in Figure 7. The meter is carefully designed to guarantee that the magnetic flux density, 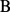, is orthogonal to the moving wires. We can use the Lorenz force on the wire and the radius of the motion of the wire, 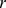, to calculate the rate of energy that is transduced per unit angle of motion:

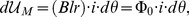
(34)where 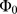 is a parameter that represents the construction of the meter. If we use multiple turns of wire then this simply re-scales the parameter, 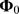, but does not alter the basic model. We can integrate Equation 34 to obtain 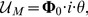
(35)which is the appropriate energy term for the Lagrangian function of a moving, current carrying coil, in a magnetic field. We note that the energy that can be transduced is unbounded, if the angle, 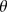, is allowed to increase without bound. Of course, this is normal for a motor. In practice the angle for the meter cannot increase outside of the range 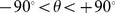 because the meter does not have a commutator. Forces would cease, and then change direction at the boundaries.

**Figure 8:**
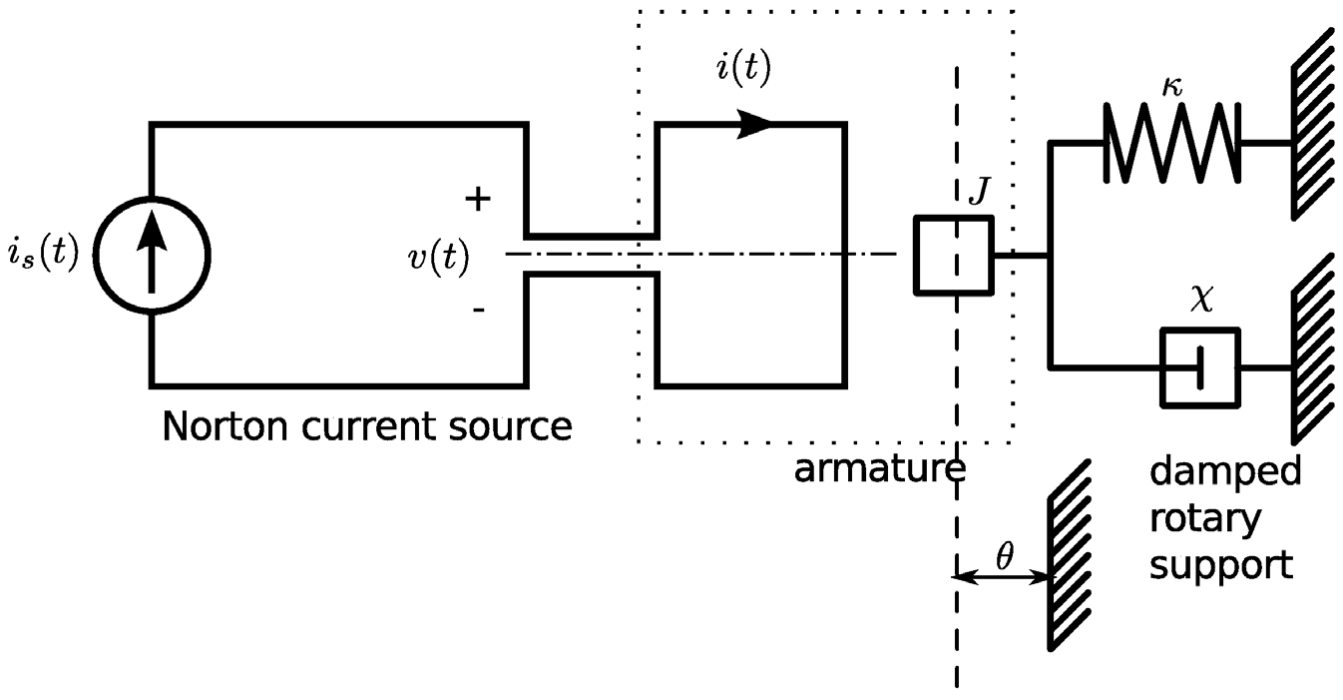
An equivalent electro-mechanical circuit for a D'Arsonval galvanometer. The Lagrangian function for this electromechanical system is written in Equation 37. The current, *i*, comes from an ideal current source, so it is essentially a constraint, rather than an independent coordinate. The last term in this Lagrangian function determines the coupling between the electrical and mechanical aspects of this system.



The moving-coil current meter is shown in Figure 7. The relevant parameters of this physical system are, the rotational moment of inertial of the coil (including the needle and the physical supports) 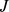, the torsional spring (stiffness) constant, 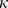, the torsional damping constant, 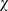 and the maximum magnetic-flux linked by the coil, 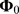, defined earlier. For a coil in free space the stored energy in the coil is given by 
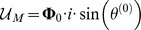
, but for the D'Arsonval and Weston style of meter the magnetic field is at right angles to a narrow circular air-gap. This makes the magnetic stored energy function more linear with respect to 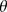. We can write 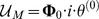, as in Equation 35. These abstract aspects of the meter and their relationships are shown in Figure 8. There is only one generalised coordinate for this system, 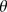 and the Lagrangian function for the linearised system can be written as

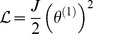


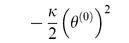


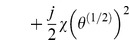


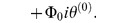
(36)


The last term represents the transduction of energy through the coil.

We can apply Equation 4 to Equation 36 and obtain the equation of motion for the D'Arsonval meter: 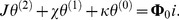
(37)


This example shows that it is possible to model mixed mechanical and electrical (electromechanical) systems using Lagrangian techniques. Further, we show that the presence of vicious damping is no obstacle to Lagrangian analysis.

## Summary and Conclusions

We have extended the range of applications of Lagrangian analysis, to include non-conservative systems that include dissipative forces. This has been achieved, even though it contradicts many of the accepted ideas in the current literature. We have also provided a systematic method of applying an extended type of Lagrangian analysis to non-conservative electromechanical systems.

The successful application of Lagrangians in dissipative, non-conserved systems depends on the appropriate substitution of variables, the choice of Legendre transformations and the use of fractional calculus of variations.

Our approach motivates a number of directions for future work:

- It is possible to extend Lagrangian techniques to non-linear dissipative systems, such as memristors or diodes, using Taylor's theorem, or by using repeated integration by parts.
- If we could extend fractional calculus of variations to include generalised functions, such as white noise, then we could develop a fractional Malliavin calculus. The greater aim is to analyse electromechanical systems in the presence of noise. We expect that this would lead to the solution of the apparent paradoxes of the Penfield motor [7], and the Davis electromechanical capacitor [43]. A complete theory should be compatible with Fluctuation Dissipation Theorem, as described by Weber [44], for example.
- Is a statistical hypothesis test due to Granger[45], which can be used to determining whether one time series is useful in forecasting another. If we had a complete theory, which could model damping forces and fluctuations, then it would be interesting to see whether Granger's sense of causality could be used to allocate a direction to the time variable.
- Extremal principles can be used to create a number of numerical methods. A number of numerical methods have recently been proposed in the literature, most notably in Almeida [25] and Pooseh [46], [47]. The opportunities for numerical solution appear to be very good. The authors have had some success using optimisation packages, such as **fincon**() in Matlab, and **sqp**() in GNU Octave. Such methods can be iterative, so an approximate solution can always be improved, through further iteration
- It is possible to apply Noether's theorem to determine the “constants of the motion” for quite general systems, including systems with dissipative elements and dependent sources. These constants of the motion will be generalised forms of momentum and energy. This is discussed in Frederico [48], [49]. the work of Kane et al. [50]is also relevant.
- For noisy electrical systems, with many degrees of freedom, it is of great theoretical interest to write down Liouville's theorem, in its most general form. The greater aim here is to understand the thermodynamics of electrical systems.

It should be possible to create a time-average Lagrangian analysis for switched-mode systems. This would be analogous to the time-averaged state-space models of Middlebrook and Ćuk [5].

In summary, we argue that the generalised Lagrangian functions described in this paper are expected to have impact on theoretical and practical applications in electrical and mechanical engineering.
